# Synthetic Biology: A Bridge between Artificial and Natural Cells

**DOI:** 10.3390/life4041092

**Published:** 2014-12-19

**Authors:** Yunfeng Ding, Fan Wu, Cheemeng Tan

**Affiliations:** Department of Biomedical Engineering, University of California Davis, One Shields Ave., Davis, CA 95616-5270, USA; E-Mails: yfding@ucdavis.edu (Y.D.); fwwu@ucdavis.edu (F.W.)

**Keywords:** artificial cells, natural cells, synthetic biology

## Abstract

Artificial cells are simple cell-like entities that possess certain properties of natural cells. In general, artificial cells are constructed using three parts: (1) biological membranes that serve as protective barriers, while allowing communication between the cells and the environment; (2) transcription and translation machinery that synthesize proteins based on genetic sequences; and (3) genetic modules that control the dynamics of the whole cell. Artificial cells are minimal and well-defined systems that can be more easily engineered and controlled when compared to natural cells. Artificial cells can be used as biomimetic systems to study and understand natural dynamics of cells with minimal interference from cellular complexity. However, there remain significant gaps between artificial and natural cells. How much information can we encode into artificial cells? What is the minimal number of factors that are necessary to achieve robust functioning of artificial cells? Can artificial cells communicate with their environments efficiently? Can artificial cells replicate, divide or even evolve? Here, we review synthetic biological methods that could shrink the gaps between artificial and natural cells. The closure of these gaps will lead to advancement in synthetic biology, cellular biology and biomedical applications.

## 1. Introduction

In 1665, Hooke observed cellular structure from cork materials and coined the word “cell”. Later, Schleiden and Schwann described cells as the basic unit of life [1,2]. Thus, “cell biology” emerged from that time and has gone through more than one hundred years of study. Despite the advancement of knowledge in cell biology, the integral functioning of cells is still not fully understood, which is likely due to the inherent complexity of natural cells. To this end, the complexity of natural cells could be overcome by building artificial cells to simplify and mimic natural cells. The concept of the artificial cell was first posed by Dr. Thomas Ming Swi Chang in 1957 [3], and it is now a pioneering and widely-known research field. Over the past few decades, extensive achievements have been made in the biotechnological and industrial applications of artificial cells [4,5,6,7,8], including co-translational insertion of membrane proteins into liposomes [9,10,11,12,13,14,15,16,17,18,19], directed evolution of cellular components [20,21], studies of primordial cells [4,22,23,24,25], delivery of drugs [26,27,28,29] and synthesis of proteins using nano-factories [30,31].

The definition of artificial cells is broad and includes various types of synthetic cells: protocells for addressing questions about the origin of life [22,23,24,25,32,33]; minimal natural cells that possess only the necessary genes for their basic maintenance [34,35,36,37,38,39]; and artificial cells that are constructed using synthetic membranes and cellular components [27,29,40,41]. These artificial cells consist of membranes that are made from lipids, fatty acids, polymers and combined lipid-polymer complexes. The artificial cells encapsulate various contents, ranging from transcription/translation machinery to multi-enzyme systems. This review focuses on artificial cells that are composed of a lipid bilayer, transcriptional/translational machinery and genetic information, with special emphasis on the application of synthetic biology in the construction of artificial cells.

The development of artificial cells indeed shares many fundamental characteristics of synthetic biology, which focuses on the minimality [22,42], modularity [43,44,45] and controllability [46,47,48,49,50] of synthetic biological systems. Taking advantage of synthetic biology, scientists could endow artificial cells with cellular functions inspired by natural systems. For artificial cells, DNA can be designed to carry information and to form genetic circuits [51,52,53,54,55]; transcription/translation machinery can be controlled under different conditions [56,57,58,59,60,61,62]; and cell membranes can be reconstituted with functional membrane proteins [9,10,11,12,13,14,15,16,18,19].

To further push the field of artificial cells, they could be engineered with the more complex characteristics of natural cells, including metabolism and autonomous replication. Artificial cells could encode for metabolic pathways using genetic circuits and cellular components [54,61]. Artificial cells could also exchange materials and information between the inside and outside of cells [24]. In addition, artificial cells could be created to replicate both the informational genome and the three-dimensional structure [4,63]. 

**Figure 1:**
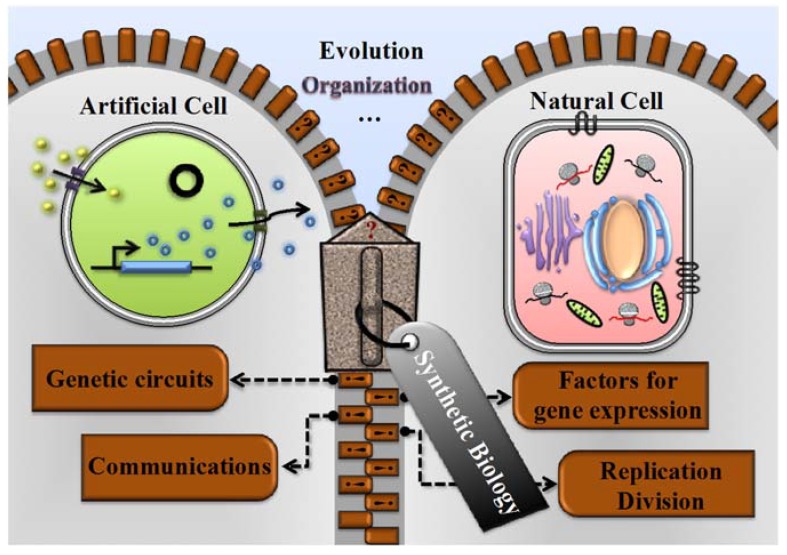
Bridging gaps between artificial and natural cells using synthetic biology approaches. For artificial cells, significant progress has been made in constructing different genetic circuits, optimizing factors for gene expression, facilitating cell-cell communication and mimicking replication. Despite the progress, there is still a gap between artificial cells (green circle) and natural cells (pink rectangle). Synthetic biology can be exploited to bridge the gap. For example, *de novo* synthesized genome DNA can be designed to encode artificial cells with more cellular functions. The natural cellular environment can be mimicked inside artificial cells to achieve efficient gene expression and signal transduction. Different membrane proteins can be reconstituted to endow the membrane of artificial cells with complex functions. Division machinery may be implemented to achieve self-replication in artificial cells.

In this review, we first depict the basic concept of artificial cellular systems. Next, we describe the gaps between artificial cells and natural cells. We then highlight the current limitations of artificial cells, as well as new challenges and opportunities in the field. Finally, we discuss the bridging of these gaps using synthetic biology approaches, focusing on genetic circuits, non-genetic factors, cell communication and self-reproduction. The filling of these gaps will eventually enable robust and efficient artificial cells (Figure 1). 

## 2. Construction of Artificial Cells

**Figure 2:**
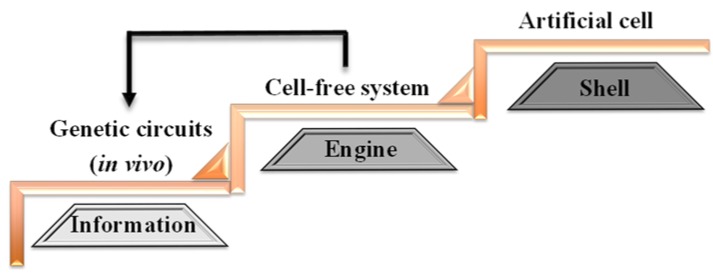
Construction of artificial cells in three steps. First step: genetic circuits are constructed *in vivo* using synthetic modules. These genetic circuits control information flow in artificial cells. Second step: the constructed circuits are tested in cell-free systems, which provide the transcription and translation engine. The feedback loop between Step 1 and Step 2 illustrates the testing and optimization of newly-constructed genetic circuits. Third Step: the circuits and the cell-free systems are encapsulated inside synthetic liposomes (the shell). The steps can be repeated in cycles to achieve optimal, efficient artificial cells.

Artificial cells are well-defined *in vitro* (or cell-free) systems that mimic certain phenotypes and functions of natural cells [4,5,23,31,32]. In general, artificial cells are made of three parts: cellular compartments (the shell), transcription and translation machinery (the engine) and genetic components (the information) [6]. Artificial cells can be constructed in three basic steps, which correspond to the three parts (Figure 2). The first step is to generate and characterize genetic circuits (the information) *in vivo*, including logic gates (e.g., AND, NOR, OR), promoters (e.g., lac, T7, *Escherichia coli* endogenous promoters), as well as different transcription factors [52,54,64]. The aim of this step is to design and test genetic circuits that give rise to the desired functions. While *in vivo* systems allow large-scale synthesis of molecular components, the genetic parts may not function *in vitro* due to differences in the operating environment, such as DNA structure [65] and molecular crowding [66,67]. Therefore, the synthesis and testing of parts are often conducted in cycles between *in vivo* and *in vitro* systems. 

The second step is to test constructed circuits in cell-free systems (the engine), because the functions of the parts might be affected by artificial chemical environments that are different from the intracellular environments of natural cells. There are two major types of cell-free systems: whole cell extracts [68] and protein synthesis using recombinant elements (PURE) systems [57]. The details of these systems can be found in other review papers [62,69]. Briefly, cell extracts are directly derived from prokaryotic or eukaryotic cytosols by removing natural cell walls, in which the exact composition of the extracts is not known. The PURE system is constructed based on purified components from *E. coli*, and the concentration of each component is tightly controlled. The aim of this step is to make sure that the synthetic machinery and circuits can function inside artificial cells.

The third step is to encapsulate the cell-free systems inside membranes (the shell), which are composed of either fatty acids or phospholipids. The shell can be constructed using the extrusion method, water-in-oil method, lyophilization method and microfluidic devices. The extrusion methods can generate artificial cells with a uniform size; however, the cellular diameter is usually limited within 1 µm, and membranes could be multi-lamellar [70]. Water-in-oil methods can produce large, unilamellar artificial cells with a heterogeneous size [71,72]. Lyophilization methods can produce large artificial cells with a heterogeneous size and lamellarity [66,73,74]. Microfluidic devices can generate artificial cells with a controllable size by adjusting the diameter of the channels and the flow speed [75]. Other factors may also affect encapsulation processes, such as the component and viscosity of cell-free systems, pH and lipid composition. These issues suggest significant room for advancing the construction of artificial cells.

## 3. Gaps between Artificial and Natural Cells

There are huge gaps between natural cells and artificial cells in the complexity of genetic materials, membrane composition and structural organization. For instance, the prokaryotic organism, *E. coli*, contains 4.6 million base pairs of DNA. The DNA encodes for 4288 annotated genes, which belong to 2584 operons [76]. These genes are translated to proteins, which yield multiple interacting partners of 2667 proteins [77]. The membranes of *E. coli* consist of three main types of phospholipids [78], which support the activity of approximately 1050 different membrane proteins [79]. The chromosome of *E. coli* is organized in specific structural domains that regulate gene expression [80]. Cell division of *E. coli* is controlled tightly by the Z ring [81] and MinCDE pathways [82]. 

In contrast to bacteria, state-of-the-art artificial cells are much simpler and composed of fewer components. In the latest work, artificial cells contain 1.77 kilo-base pair DNA (coding sequence for functional proteins), which encode for two genes [73]. The highest number of functional proteins included inside artificial cells is three [83] (excluding machinery that support transcription and translation). Crowded environments inside artificial cells are created by adding crowding agents, including PEG, ficoll and dextran [84]. For each artificial cell, its membrane is typically reconstituted using one to two types of phospholipids [15,73] and a maximum of one type of pore-forming protein [71]. Significant progress has been made in the construction of artificial cellular systems, such as encapsulation of different genetic circuits, incorporation of natural and non-natural components and assembly of natural and synthetic membranes [4,5,32,61,75]. On the one hand, these achievements have seemingly closed the gaps between prokaryotic cells and artificial cells by establishing the basic structure of cells, which include membranes, transcription-translation machinery and genetic pathways. On the other hand, the gaps between natural cells and artificial cells are constantly increasing due to the rapid discovery of new mechanisms in simple prokaryotic cells, including RNA localization [85,86] and CRISPR-based defense against phages [87]. The gaps between prokaryotic and artificial cells bring tremendous opportunity to improve artificial cells by exploiting new concepts and tools in synthetic biology. 

### Synergy between Synthetic Biology and Studies of Artificial Cells

Synthetic biology is a field that focuses on using well-defined genetic parts to build new synthetic systems. One of the goals is to have the capacity to design and build synthetic cells with predictable functions and applications, including the production of biocommodities, therapeutic treatment and biosensors [88,89]. Indeed, recent reviews of synthetic biology have highlighted artificial cells as promising synthetic systems [88,89]. The field approaches biological engineering from several unique angles. First, synthetic biology highlights design principles that can guide the control of biological systems [90,91]. Rational design is a mainstay of synthetic biology that relies on the idea that biological systems are fundamentally modular [54,92]. During the design process, genes are defined as the basic biological units. The aim of rational design is to generate an optimized outcome through logical assembly of these basic units. For example, the synthesis of the precursor of the anti-malaria drug, artemisinic acid was achieved by coupling an engineered mevalonate pathway with two enzymes (amorphadiene synthase and cytochrome P450 monooxygenase) in yeast [93].

Second, synthetic biologists use well-characterized and interoperable modules, such as promoters, operators, transcriptional factors and ribosome binding sites, as building blocks to create higher-order circuits [53,94,95,96,97]. For example, a promoter library was used to ascertain rules that describe the responsiveness of a promoter to transcriptional factors [98]. Specifically, promoters were sub-divided into the core, proximal and distal regions. For prokaryotes, the strength of transcriptional repression was shown to be the greatest when a repressor site was located in the core region of a promoter. The repression strength was less strong when located in the proximal region and was the weakest when located in the distal region. Conversely, activators worked only in the distal region and had no effect in the core and proximal regions [99]. These basic principles could be exploited to assemble promoter modules in a bottom-up approach.

Third, synthetic biology uses a bottom-up approach to understand biological circuits. One can design and construct simple genetic circuits from well-characterized genes and proteins, followed by the analysis of their behavior in living cells. Through this approach, tremendous insights are gained into noise propagation [100,101,102,103], network motifs [104] and the dynamics of nonlinear genetic circuits [105,106,107,108,109,110]. In addition, DNA synthesis is a powerful tool for large-scale synthesis of genetic circuits. A state-of-the-art DNA synthesis method that employs error correction reaction can achieve a low error rate of one in 8700 base pairs [111]. Such DNA synthesis has been exploited for metabolic engineering [112] and genome construction [113,114].

Fourth, synthetic biology provides a computational toolbox to model synthetic systems. Modeling is useful to ensure that the assembled systems operate as desired. Modeling is also useful for suggesting the specific manipulation of system components. For example, a thermodynamic model was developed to predict protein expression by designing various ribosome binding sites (RBS). To test the model, designed RBS sequences were connected to the P_BAD_ promoter and an AND-gate circuit to optimize green fluorescent protein (GFP) expression [115]. *In silico* modeling based on libraries of diversified components was used to design a synthetic gene network that functioned as a timer [51].

The engineering of artificial cells shares many characteristics of synthetic biology approaches. Both biological and non-natural building blocks can be used to create genetic circuits in artificial cells [116,117,118,119,120,121]. For example, incorporation of non-natural nucleic acids (e.g., XNA, PNA) can increase the resistance of nucleic acids to nucleases and improve binding specificity between two nucleic-acid strands [122,123]. Non-natural amino-acid incorporation will expand the structural and functional diversity of proteins [124,125]. Nanotubes/pores can be used as membrane channels for the transport of biological and inorganic molecules [126,127,128]. In the following sections, we will highlight four areas to apply concepts from synthetic biology toward the construction of artificial cells, including genetic circuits, non-genetic factors, communication and replication.

## 4. Using Synthetic Biology to Shrink the Gaps between Artificial and Natural Cells

### 4.1. The Design of Genetic Circuits to Control Functions of Artificial Cells

Synthetic biologists can now rewire natural cellular networks by either constructing orthogonal genetic circuits or modifying endogenous circuits of the host cells [52,54,129,130]. For example, anti-malaria drug was synthesized by increasing farnesyl pyrophosphate production, introducing an amorphadiene synthase gene and incorporating a novel cytochrome P450 and its redox partner [93]. These methods of designing and constructing synthetic gene circuits could be exploited for the control of artificial cells. 

Two main requirements are required to endow artificial cells with more complex genetic circuits. The first requirement is the ability to synthesize long DNA, which carries more information than a short fragment of DNA. The continuous improvement in *de novo* DNA synthesis and gene assembly technologies has enabled the synthesis of DNA at mega-base pair scale with high accuracy [131,132]. The J. Craig Venter Institute synthetic 1.0 genome is 1.08 million base pairs in length and contains about 430 genes [49]. Furthermore, a functional eukaryotic chromosome (272,871 base pairs) has been synthesized in a stepwise manner [133]. Therefore, DNA synthesis is a powerful tool to design long DNA for implementation in artificial cells.

The second requirement involves the design of genetic circuits for controlled gene expression. A classical engineering procedure could be implemented to fulfill this requirement: understanding, design, and analysis [134]. First, engineers set clear objectives for the intended design of genetic circuits. Next, to accomplish the objectives, computational algorithms are used for genetic design [135] and network wiring [136]. To this end, diverse datasets have been integrated to build a database of essential genes and to design metabolic pathways and signal transductions [137,138,139,140]. Computational tools can also be used to predict expression levels of selected genes for the better construction of cellular networks in artificial cells [141]. Third, system analysis is performed according to the desired outcomes. A new round of the engineering procedure is initiated until the desired outcomes are achieved. When compared to natural cells, the simplicity of artificial cells allows precise control of desired cellular phenotype and behavior. In addition, it is now plausible to synthesize genomic DNA for the construction of artificial cells. One of the future challenges will be the true design and engineering of a synthetic genome without any reference templates [131].

**Figure 3:**
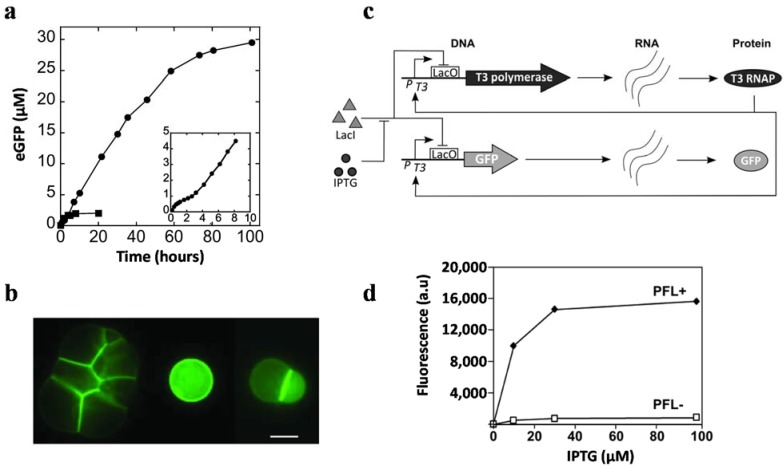
(**a**) Kinetics of α-hemolysin-eGFP expression. The presence of α-hemolysin (filled circles) prolonged the expression of eGFP from ~20 h to days. Filled circles: 0.5 nM pIVEX2.3d-α-hemolysin-eGFP. Filled squares: the expression of eGFP inside liposomes without α-hemolysin. The inset indicates the first 10 h of gene expression. (**b**) The *E. coli* extract was encapsulated in vesicles with pIVEX2.3d-α-hemolysin-eGFP surrounded by feeding solution. Expression of α-hemolysin-eGFP was observed in aggregate vesicles (**left**), single vesicle (**middle**) and doublet (**right**) (scale bar, 20 μm) (reprinted with permission from [71], Copyright 2004, The National Academy of Sciences). (**c**) Schematic diagram of a positive feedback loop (PFL). The T3 RNA polymerase (T3 RNAP) gene was regulated by T3-lacO promoter. The addition of IPTG induced T3 RNAP expression. The T3 RNAP promoted its own transcription and activated GFP expression. (**d**) Comparison of GFP expression with or without a PFL. DNA fragments (3 nM) shown in (c) were mixed with a cell-free system containing purified LacI and T3 RNAP. GFP expression was measured at 180 min after the addition of IPTG. The signal-to-noise ratio was increased from 75 to 800 with the PFL (reprinted with permission from [83], Copyright 2013, Royal Society of Chemistry.)

To date, DNA [142], RNA [143] and peptides [70] have been synthesized in liposomes using synthetic gene circuits. The first example of DNA amplification was implemented through polymerase chain reaction (PCR) inside liposomes. The liposomes were stable at high temperature conditions used for the PCR [142]. Template-independent RNA polymerase was encapsulated inside dimyristoyl phosphatidylcholine vesicles. Long chain RNA was synthesized when adenosine diphosphate (ADP) was externally provided [143]. The synthesis of functional GFP was the first successful attempt at protein expression inside liposomes [144]. Based on this foundation, continuous synthesis of enhanced GFP (eGFP) inside artificial cells was achieved by incorporating α-hemolysin in the membranes. These artificial cells could sustain protein production for up to four days (Figure 3a,b) [71]. A two-stage genetic network was constructed in liposomes using two different plasmids. In this study, SP6 promoter regulated the expression of T7 RNA polymerase (T7 RNAP) in one plasmid, and the T7 RNAP regulated GFP expression through a T7 promoter in another plasmid [74]. A positive feedback loop was introduced into artificial cells and was shown to increase the signal-to-noise ratio to 800 when compared to circuits without the positive feedback loop (Figure 3c,d) [83].

However, gene circuits constructed in artificial cells are still limited to a few genes. How much information do we need to encode into an artificial cell to mimic a minimal cell? The minimal genome refers to a set of genes that are required to maintain life [34,35,36,37,38,39]. To date, the smallest genome is predicted to be 113 kilo-base pairs long and contains 151 genes. These genes include 38 RNAs and 113 proteins that form the core cellular replication machinery [42]. We envision that such a minimal genome could be incorporated into artificial cells to establish the foundation of free-living artificial cells. The incorporation is non-trivial, as it requires understanding all genetic and non-genetic factors that modulate critical cellular functions. 

### 4.2. Non-Genetic Factors That Modulate Gene Expression in Artificial Cellular Systems

In addition to the genome, natural cells are regulated by various non-genetic factors, which could be harnessed to improve the control of artificial cells. DNA structure is one of the non-genetic factors that modulates gene expression [145]. For example, a poly (dG)-poly (dC) sequence was used to form different lengths of a DNA triplex. This non-B-form DNA structure modulated gene expression when placed at the 5’ end of a promoter. The activity was length dependent: the sequence affected the expression of reporter genes when placed 27–30 base pairs upstream of the promoter, but exhibited no effects when placed further than 35 base pairs upstream of the promoter [146]. Indeed, DNA structures have been exploited to control synthetic biological systems. For example, single-strand DNA was engineered as scaffolds to form extracellular matrix with proteins. In this work, the persistence and stiffness of the DNA scaffold were controlled by adding single-stranded domains. This kind of extracellular matrix could affect cytoskeletal arrangement and cellular shape, as well as signal transduction [147]. Therefore, the DNA structure is a potential tool for controlling the dynamics of artificial cells.

Non-DNA binding factors represent another class of non-genetic factors that regulate gene expression. Non-DNA binding factors refer to factors that affect gene expression, but do not directly bind to DNA molecules. For example, *osmZ* (also known as *hnsA*) can increase the DNA supercoil in bacteria [148]. Mutation of this gene affects the expression of *ompF*, *ompC*, *fimA* and *bgl* operons [149]. A histone-binding protein, nucleoplasmin, binds to histone and reduces its affinity to DNA, which, in turn, increases the binding probability of transcription factors (GAL4-AH, USF, Sp1) to DNA [150]. Histone acetyltransferase and deacetylase are responsible for histone acetylation and de-acetylation that play causative roles in gene transcription [151]. These non-DNA binding factors could be exploited to improve the control of gene expression inside artificial cells.

In addition, molecular crowding is another non-genetic factor that modulates gene expression [65,66,67,84,152,153,154,155,156,157,158,159,160]. The cytosol of natural cells consists of highly-packed macromolecules, including proteins, nucleic acids, carbohydrates and ribosomes. The typical concentration of these macromolecules is 300–400 mg/mL [152]. This molecular crowding has been shown to limit the diffusion of macromolecules and to enhance their interactions *in vitro*. Furthermore, molecular crowding enhances the stability of DNA [161] and proteins [162,163], affects the diffusion of transcription proteins [153,164,165,166] and promotes self-assembly of macromolecules [167]. At present, whole cell extracts and PURE systems are commonly used to provide the machinery for transcription and translation in artificial cells. The most common crude lysates are derived from three sources, including *E. coli* extract [68], wheat germ extract (WGE) [168] and rabbit reticulocyte lysate (RRL) [169]. Protein concentration in bacterial extracts is approximately 10 mg/mL, which is 30-fold lower than the protein concentration in living cells [4].

**Figure 4:**
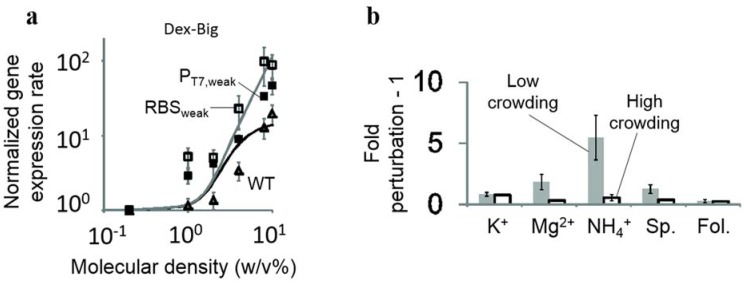
The effect of molecular crowding on gene expression. (**a**) Gene expression rates in environments containing big crowding agent (Dextran-Big). The reporter gene, cyan fluorescent protein (*cfp*), was under the control of a normal T7 promoter (P_T7_), a weak T7 promoter (P_T7, weak_) or a weak ribosome binding site (RBS_weak_). The black line represents the predicted expression rates of *cfp* from normal P_T7_. The grey line represents the predicted expression rates of *cfp* from weak P_T7_. Experimental data (open triangles for WT, open squares for RBS_weak_, filled squares for T7_weak_) follow the prediction. (**b**) Perturbation of gene expression rates using different concentrations of potassium glutamate (K^+^), magnesium acetate (Mg^2+^), ammonium acetate (NH_4_^+^), spermidine (Sp.) and folinic acid (Fol.). Gene expression was less perturbed in highly-crowded environments (black open bars) than that in low crowded environments (grey bars) (reprinted with permission from [66], Copyright, 2013, Nature Publishing Group).

To mimic molecular crowding, crowding agents that are inert macromolecules, such as PEG, dextran and ficoll, can be supplemented in cell-free systems [66,170]. A recent work mimicked crowded environment by inducing coacervation in *E.coli* lysate using osmosis pressure. The study showed that transcription rates were five- to six-times higher in a crowded environment than non-crowded conditions [171]. Tan *et al.* [66] introduced molecular crowding to artificial cellular systems and found that the binding of RNA polymerase to the promoter was increased by a large crowding agent. They also found that molecular crowding enhanced the robustness of gene expression under chemical perturbations (Figure 4). Recently, an additive-free cell extract (AFCE) was used to construct life-mimicking artificial cells (L-MACs). Specifically, a semi-permeable membrane was used to condense the extract to 260 mg/mL of macromolecules, which was close to the protein concentration in living cells. However, protein expression was low in L-MACs, suggesting that simple condensation of the bacterial extract gave rise to sub-optimal conditions for gene expression [172]. Importantly, this work suggests that active, unknown cellular pathways may be necessary for the modulation of crowding conditions inside natural and artificial cells for optimal gene expression.

### 4.3. Communication between Artificial Cells and Their Environment

Living cells communicate with their surroundings to adapt to changing environments. The communication is achieved through cell membranes that control signal transduction, energy production and trans-membrane channels for molecular transport. Along this line, components of the natural cell membrane could be incorporated into artificial cells, which would transform them from passive entities into active systems that can interact with environments. 

**Figure 5:**
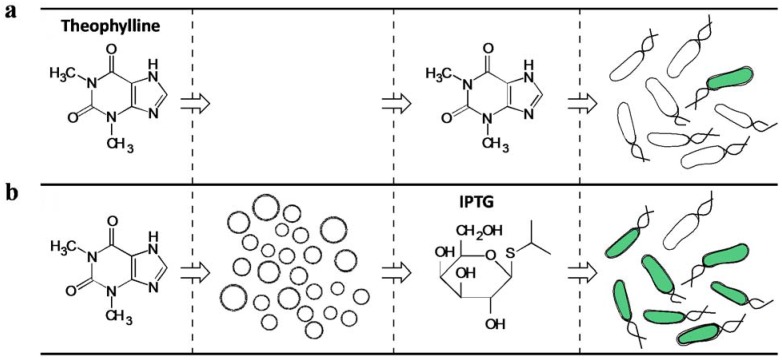
Artificial cells translate chemical signals for *E. coli*. (**a**) Theophylline cannot diffuse through the cell membranes of *E. coli*. Without artificial cells (circles), *E. coli* cannot sense theophylline. (**b**) Theophylline can diffuse through artificial cell membranes. Artificial cells sense theophylline and express α-hemolysin to form unspecific pores on their membranes. The entrapped IPTG is released to trigger GFP expression in *E. coli* (reprinted with permission from [73], Copyright 2014, Nature Publishing Group).

The simplest way for artificial cells to sense the environment is through small molecules that directly permeate membranes. For example, theophylline was used as a signal that diffused through artificial cell membranes and directly bound to mRNA to turn on yellow fluorescent protein (YPet) expression [173]. Based on this communication mechanism, Lentini and colleagues implemented artificial cells that translated chemical signals for *E.coli*. In this work, theophylline could not be recognized by *E. coli*, but would permeate into artificial cells and turn on α-hemolysin expression through a theophylline riboswitch. The α-hemolysin formed an unspecific pore that allowed the transition of isopropyl β-D-1-thiogalactopyranoside (IPTG). The entrapped IPTG was released from artificial cells to trigger gene expression in *E. coli*. This work represents the first example of cross-species communication between artificial and natural cells (Figure 5) [73]. Despite the successful implementation, the authors encountered an issue with leaky expression of α-hemolysin, which caused gene expression in *E. coli* to eventually converge for systems with and without the input signal, theophylline. This issue suggests significant room to improve the control of synthetic modules *in vitro*. 

**Figure 6:**
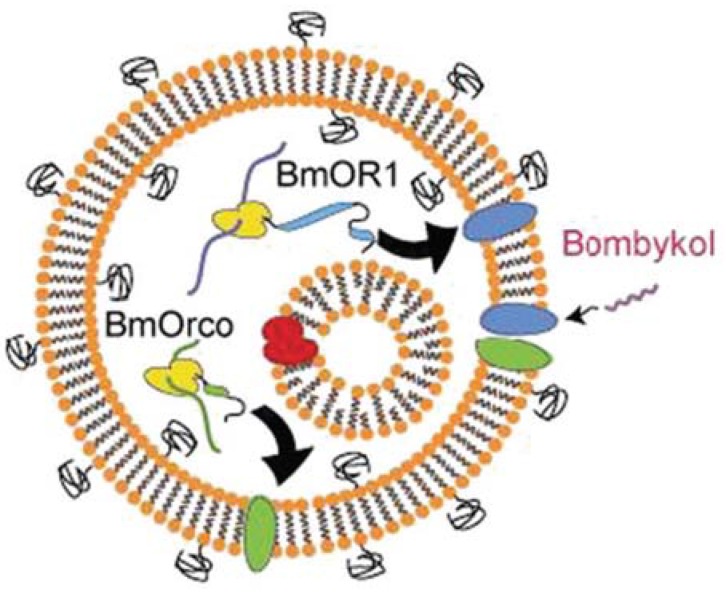
Schematic diagram of reconstituted olfactory receptor. The expression circuits of the olfactory receptor (BmOR1) and its co-receptor (BmOrco) were constructed inside giant vesicles (GVs). Canine pancreatic microsomal membranes (the small vesicle inside the GV) were added inside GVs to promote cell-free synthesis. BmOR1 and BmOrco were expressed and inserted into the GV membranes to form a complex. The olfactory complex was stimulated by its ligand, bombykol. The effect of stimulation can be detected by a voltage clamp (reprinted with permission from [72], Copyright 2014, Royal Society of Chemistry).

Artificial cell membranes can also be modified using membrane proteins to perform certain functions. For example, α-hemolysin was expressed and inserted into artificial cell membrane. It formed unspecific pores on the cell membrane, which allowed molecules smaller than 3 kDa to diffuse through the membranes [71]. Cytochrome b5 and its fusion proteins were synthesized and directly localized on liposome membranes [9]. Potassium channel KcsA was introduced into the lipid bilayers of artificial cells with a controllable orientation [13]. Functional Sec translocon machinery were localized in the membranes of artificial cells [16]. BmOR1 and BmOrco that formed an olfactory receptor complex were incorporated into liposomes to detect the ligand, bombykol (Figure 6) [72]. These studies provide examples to reconstitute membrane proteins in artificial cell membranes, which could be used as modules to construct communicating artificial cells.

### 4.4. Replication and Division of Artificial Cells

Recent work has demonstrated that artificial cells could grow by incorporating micelles, which provided additional phospholipids for the growth of cell membranes. Division of artificial cells was then achieved by agitation [25,174,175]. To date, artificial cells cannot self-replicate autonomously without external intervention [4]. The challenge of autonomous replication arises due to the difficulty of reconstituting both metabolism and division machinery inside artificial cells. Attempts have been made toward supporting metabolism inside artificial cells. To enable the consistent supplement of nutrients, α-hemolysin was incorporated into artificial cell membranes. Based on the approach, gene expression was sustained inside artificial cells for up to four days (Figure 3a,b) [71].

**Figure 7:**
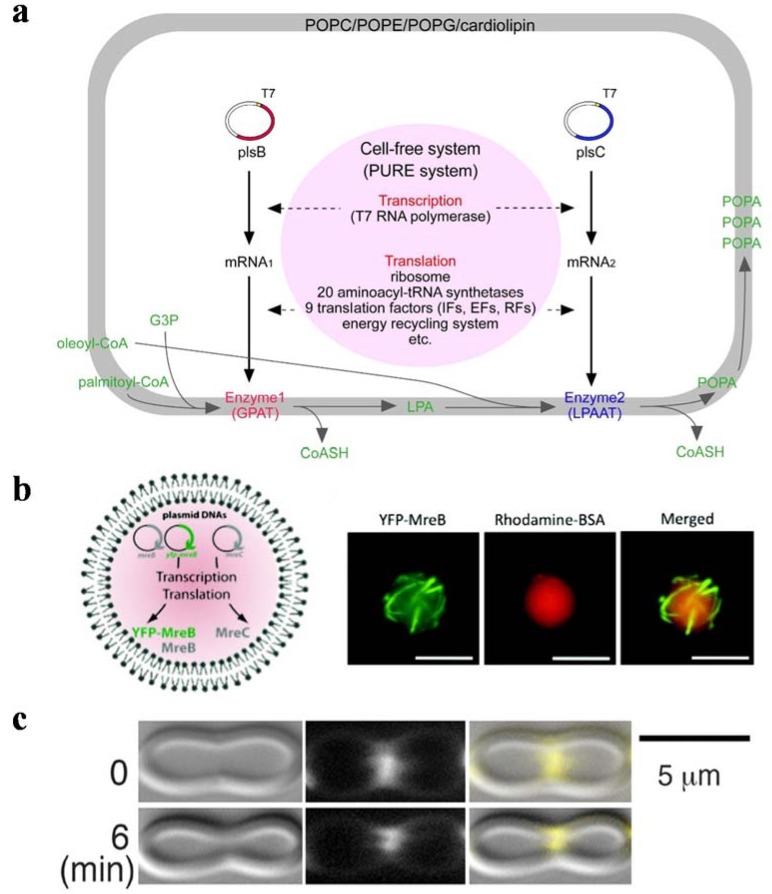
Basic elements and processes for self-replication of artificial cells. (**a**) Schematic of artificial cells with the capability of lipid-synthesis. Sn-glycerol-3-phosphate acyltransferase (GPAT) catalyzed glycerol-3-phosphate (G3P) to form lysophosphatidic acid (LPA). Lysophosphatidic acid acyltransferase (LPAAT) generated phosphatidic acid (PA) using LPA. GPAT and LPAAT were expressed in artificial cells to sustain PA synthesis (reprinted with permission from [19], Copyright 2009, Elsevier.) (**b**) Schematic of MreB, YFP-MreB and MreC expression in artificial cells (**left**). Co-expression of MreB, YFP-MreB and MreC inside artificial cells, which were imaged by a microscope (**right**). The fluorescence of YFP (green pseudocolor) showed that MreB formed a filamentous structure in the presence of MreC. Rhodamine (red pseudocolor) showed that artificial cells were isolated from the extracellular feeding solution (scale bar, 10 μm) (reprinted with permission from [176], Copyright 2012, American Chemical Society). (**c**) The tubulin-like protein, FtsZ, formed a Z ring on the artificial cell membrane. (**Top**) Z rings were first observed to form a constriction on the artificial cell (0 min). (**Bottom**) A more obvious constriction formed after 6 min (scale bar, 5 μm) (reprinted with permission from [177], Copyright 2008, The American Association of the Advancement of Science).

Other findings have provided preliminary results for reconstituting cell division machinery inside artificial cells. To approach autonomous division, the phosphatidic acid (PA) synthesis pathway was reconstituted inside liposomes, which generated functional sn-glycerol-3-phosphate acyltransferase (GPAT) and lysophosphatidic acid acyltransferase (LPAAT) for membrane growth (Figure 7a) [19]. In addition, bacterial cytoskeleton MreB was reconstituted into lipid membrane and shown to exhibit filament structures (Figure 7b) [176]. FtsZ was demonstrated to form Z rings in liposomes and to generate a force for fission (Figure 7c) [177]. To this end, elementary steps of natural cell division have been reconstituted inside artificial cells. However, significant work is required to connect these steps for integral functioning of autonomous, artificial cell replication.

Instead of reconstituting the replication machinery of complex cells, the machinery of simpler organisms, such as phages and viruses, could be exploited for the construction of artificial cells. Indeed, T7 and encephalomyocarditis virus (EMCV) were shown to replicate and assemble themselves in whole cell extracts of prokaryotes [178] and eukaryotes [179]. The 40 kilo-base pair T7 genome DNA was incubated in *E. coli* whole cell extract that contained nutrients for transcription and translation at 29 °C for 12 h. Phage replication in the reaction mix was measured by counting plaque forming units. More than a billion infectious T7 phage per milliliter were generated. Similarly, the 7.1 kilo-base pair EMCV genome DNA was assembled in a T7 promoter/terminator unit and incubated in a HeLa cell extract containing T7 RNAP. The plaque forming unit was determined by using BHK-21 cells and reached eight billion particles after 8 h of incubation at 34 °C. These studies suggest a potential direction for constructing artificial cells following phage replication pathways.

## 5. Conclusions and Future Outlook

In 1925, Gorter and Grendel presented the first evidence that cellular membranes are composed of lipid bilayers [180]. Today, we have reached a critical barrier in the research of artificial cells that consist of bilayer membranes and protein synthesis machinery. Can we implement the minimal set of components required to create free-living artificial cells? Answers to the question will challenge our basic understanding of natural cells and emergent properties of complex systems. Based on studies of free-living natural cells, *Mycobacterium genitalium* has the smallest genome size of 580,076 base pairs that contain only 475 coding sequences [181]. The coding sequences include genes required for DNA replication, transcription and translation, DNA repair, cellular transport and energy metabolism. Indeed, due to the minimality of the genome, it was the first genome to be synthesized chemically and used to create the first synthetic *Mycobacterium mycoides* [49]. In this work, a chemically-synthesized genome was inserted into natural *Mycobacterium capricolum* that was stripped of its original genome. Essentially, the synthetic genome was used to reboot the bacteria by using existing cellular proteins and structures. Can a similar strategy be used to insert synthetic genomes into artificial cells?

To this end, synthetic biology has established a rich library of tools and cellular parts, which could be exploited to achieve free-living artificial cells. Pathway databases and automated design tools could be used to design and predict cellular pathways inside artificial cells [182,183,184,185]. High-throughput cloning, gene synthesis and assembling of pathways could be used to rapidly investigate a large set of candidate pathways [186,187,188,189,190]. In addition, random DNA mutation and microfluidics could be used to evolve a large library of DNA for implementation in artificial cells [75,191,192,193]. Despite the availability of these tools, significant research is required to identify the minimal set of cellular parts required for efficient metabolism and replication [37,39,42]. Furthermore, the understanding of how intracellular non-genetic factors, including crowding, chemical species and cellular structures, regulate efficient functioning of cellular pathways is lacking [4,5,6,22]. The minimal cellular parts and non-genetic factors together constitute the minimum physical genome that is necessary to construct free-living artificial cells. While the realization of free-living artificial cells is still far from current technology, the progress toward this ultimate goal will likely reveal tremendous insights into fundamental principles that govern robust and efficient functioning of natural cells.

Artificial cellular systems are emerging as potential biotechnological systems due to their capability of mimicking certain cellular functions *in vitro*. We envision that further development of genetic circuits, non-genetic modules, cell-cell communication and self-replication will enhance the control and implementation of artificial cells. Artificial cells could be built module-by-module using nucleic acids, lipids, proteins and other molecules essential for life [97]. One day, it may be possible to create modular toolkits for computer-guided design of artificial cells, which will represent a new class of synthetic cells. 
